# Twist-Induced Beam Steering and Blazing Effects in Photonic Crystal Devices

**DOI:** 10.1038/s41377-025-01942-7

**Published:** 2025-08-07

**Authors:** Nicolas Roy, Beicheng Lou, Shanhui Fan, Alexandre Mayer, Michaël Lobet

**Affiliations:** 1 Namur Institute for Complex Systems, University of Namur, Namur, 5000 Belgium; 2 Namur Institute for Structured Matter, University of Namur, Namur, 5000 Belgium; 3 Department of Applied Physics and Ginzton Laboratory, Stanford University, Stanford, California 94305 USA; 4 John A. Paulson School of Engineering and Applied Sciences, Harvard University, 9 Oxford Street, MA-02138 Cambridge, USA

**Keywords:** Photonic crystals, Photonic devices

## Abstract

Twisted bilayer photonic crystals introduce a twist between two stacked photonic crystal slabs, enabling strong modulation of their electromagnetic properties. The change in the twist angle strongly influences the resonant frequencies and available propagating diffraction orders with applications including sensing, lasing, slow light or wavefront engineering. In this work, we design and analyze twisted bilayer crystals capable of steering light in a direction controlled by the twist angle. To achieve beam steering, the device efficiently routes input power into a single, twist-dependent, transmitted diffraction order. The outgoing light then follows the orientation of this diffraction order, externally controlled by the twist angle. Our study shows, using systematic exploration of the design space, how the device resembles blazed gratings by effectively canceling the undesired diffraction orders. The optimized devices exhibit a shared slant dependent on the selected diffraction order and that proves robust to the twist angle. Our analysis is supported by a classical blazing model and a data-oriented statistical analysis. The data-oriented approach is steered by high-efficiency heuristic optimization method, which enabled the design of optimized devices demonstrating an efficiency above 90% across twist angles ranging from 0 to 30° for both TE and TM polarizations. Extending the optimization to include left- and right-handed polarizations yields overall accuracy nearing 90% when averaged across the entire 0 to 60° control range. Finally, with the identification of the blazing effect in this initially black box structure, we show one can consider simpler design for a first prototype.

## Abstract (web summary)

We propose high-performance, tunable twisted bilayers for beam steering. An analytical model reveals their operation principles, enabling the design of simplified, efficient device candidates.

## Graphical abstract

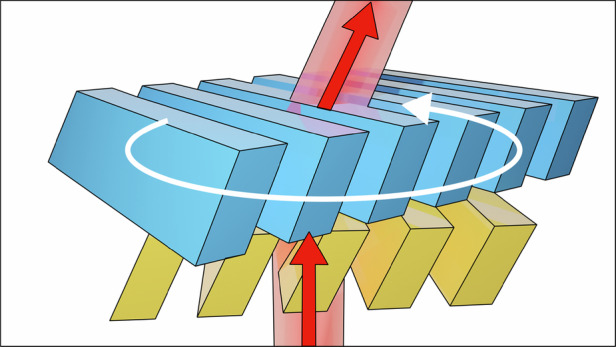

## Introduction

Twisted bilayer photonic crystals have garnered increasing attention due to their ability to introduce twist-dependent resonant frequencies, symmetry breaking, and control over their rich reciprocal lattice extended by the Moiré wavevectors^1,2^. Associated remarkable phenomena include optical flat bands^3,4^, energy localization^5,6^, optical singularities^7^, and lasing^8–10^. The bilayer implementation enables on-chip reconfiguration of the twist for various optical applications, including beam shaping^11^, sensing^12,13^, tunable circular dichroism^14^, and frequency filtering^15^. Additionally, microelectromechanical systems (MEMs) offer an effective solution for this reconfiguration^16,17^.

Beam steering refers to the control of the direction of a light beam. In photonic systems, beam steering can be achieved by active metasurfaces^18^, optical phased arrays^19,20^, or cascaded dielectric bilayers^21^. Recently, twisted bilayer photonic crystals were shown to be a promising approach by achieving efficient beam steering with a pair of dielectric layers only a few wavelengths thick^22^. Indeed, by adjusting the dielectric distribution within the layers, one can engineer diffraction efficiency^23^. While widely used blazed gratings operate on this principle, they lack tunability^24^. In contrast, the proposed twisted bilayer photonic crystal device routes input power into a single twist-dependent diffraction order, resulting in a reconfigurable output beam. Here, the beam direction is directly controlled by the twist angle between the two layers. By emitting in a single diffraction order, the device also inherits advantages of blazed gratings: they offer better beam control than phased arrays that actively manipulate the phase of multiple emitters. These arrays of emitters with a typical pitch larger than the wavelength, introduce multiple diffraction orders. Furthermore, the twisted device’s active surface is theoretically boundless and does not emit light itself^25^, allowing for seamless integration into on-chip systems. However, engineering such dielectric photonic crystals is a complex challenge. The twist angle adds an extra dimension of complexity to the design because the device lattice itself can change during operation. The freeform inverse design approach used for twisted bilayers^22^ has led to intricate but high-performing designs, although their operational framework is not yet fully understood. Is the device’s performance primarily attributable to the complex dielectric engineering, or is there a more fundamental operational principle at play? Addressing this question is crucial for advancing the development of any beam steering device based on twisted bilayer photonic crystals.

This work aims to address the aforementioned question. Therefore, we provide herein a thorough theoretical analysis augmented by high-performance optimizations to design efficient beam steering devices. Three templates of devices are explored: mini-layers^22^ and (in) a homogenous combination of dielectric ellipses. Our analysis is grounded in data-driven methodologies, leveraging high-throughput simulations^26^ to explore the design space effectively. Guided by particle swarm optimization (PSO)^27–29^, we reveal that these optimized designs exhibit a consistent blaze angle tailored to the selected diffraction order. Notably, our findings demonstrate that optimal devices mostly rely on a blazed configuration to suppress undesired diffraction orders. This point is further supported by an analytical model of the device. These insights pave the way for advancing the design and fabrication of beam-steering twisted bilayer photonic crystals which could capitalize on established expertise in blazed gratings.

## Results

**Fig. 1:**
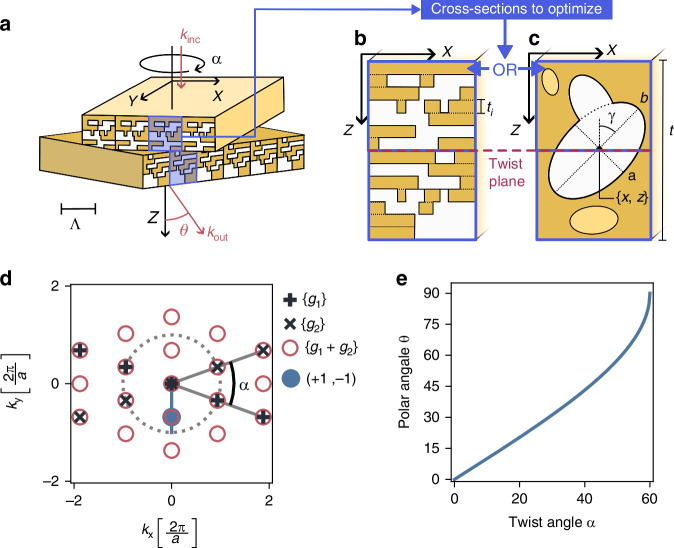
Device description. **a** Illustration of the twisted bilayer photonic crystal used for beam steering. **b**, **c** Gratings and ellipses geometrical parameterizations are illustrated. **d** The diffraction orders of both layers and those resulting from the twisted bilayer structure with an emphasis (blue dot and trajectory) on the targeted order in this work. **e** The polar angle *θ* of the targeted order wavevector varying with a range of the control i.e., twist angle α

The device, depicted in Fig. 1a, comprises two misaligned photonic crystal slabs stacked in free space. These two crystals comprise of a stack of 1D gratings. This configuration allows for a tunable diffraction pattern with the misalignment (twist) angle $\alpha$. We will be looking for orders that have twist-dependent orientation. One-dimensional gratings are sufficient for getting this effect and limit both modeling and fabrication complexity. Each slab is patterned with a unit cell of pitch Λ in the ${xz}$-plane, following different templates illustrated in Fig. 1b, c. These unit cell patterns are then extruded along the *y*-axis to obtain the layers. Given that these crystals are 1D PhC slabs, they possess the following reciprocal vector sets

$$\left\{{{\boldsymbol{g}}}_{{\bf{1}}}\right\}=\left\{R\left({\alpha }_{1}\right)i\frac{2\pi }{\Lambda }{\boldsymbol{x}},\forall i\,{\mathbb{\in }}\,{\mathbb{Z}}\right\}{and}\left\{{{\boldsymbol{g}}}_{{\bf{2}}}\right\}=\left\{R\left({\alpha }_{2}\right)j\frac{2\pi }{\Lambda }{\boldsymbol{x}},\forall j\,{\mathbb{\in }}\,{\mathbb{Z}}\right\}$$

with $R\left(\alpha \right)$ a rotation matrix and ${\boldsymbol{x}}$ a unit vector along the *x* direction. Here, we consider a twist $\alpha$ between the layers by taking ${\alpha }_{1}=-\frac{\alpha }{2}$ and ${\alpha }_{2}=\frac{\alpha }{2}$. The resulting twisted bilayer forms a hexagonal unit cell, with its truncated reciprocal space illustrated in Fig. 1d as red circles. More generally, the set of reciprocal space vectors is

$$\left\{{{\boldsymbol{g}}}_{{\bf{1}}}+{{\boldsymbol{g}}}_{{\bf{2}}}\right\}=\left\{\left(i+j\right)R\left(\frac{\alpha }{2}\right)\frac{2\pi }{\Lambda }{\boldsymbol{x}}+\left(j-i\right)R\left(-\frac{\alpha }{2}\right){\boldsymbol{y}},\forall i,j\in {{\mathbb{Z}}}^{2}\right\}$$

with ${\boldsymbol{x}}$ and ${\boldsymbol{y}}$ the unit vectors along *x* and *y* directions. Below we denote each element in the set, which corresponds to a diffraction order, as $\left(i,j\right)$. The design of the unit cell focuses on concentrating diffraction efficiency into a single Moiré diffraction order $\left(+1,-1\right)$, as represented by the blue dot in Fig. 1d. The targeted order could be both $(+1,-1)$ or $(-1,+1)$ as these exhibit twist-dependent magnitudes. The magnitude of each reciprocal lattice vector g directly affects the associated out-of-plane wavevector component

1 $${k}_{z}=\sqrt{{\left(\frac{2\pi }{\Lambda }\right)}^{2}-{g}_{x}^{2}-{g}_{y}^{2}}$$

influencing light propagation. Orders like (0,0), (0,1), and (1,0) are unsuitable because their magnitudes are either zero or invariant with respect to α. Although higher diffraction orders can exhibit similar steering capabilities, we focus on lower orders because a suitable choice of *λ* relative to Λ renders higher orders evanescent, as implied by Eq. 1. Such a situation is met when ${k}_{z}$ is a complex number, which arises when

2 $$\Lambda \le \lambda$$

This strongly reduces the number of available orders in both reflection and transmission media, keeping only three diffraction orders (0,0), (−1,1), and $(+1,-1)$ in the light cone of Fig. 1d. Tunable steering in $(+1,-1)$ is achieved as the outgoing beam inherits the polar angle dependency on the twist angle shown in Fig. 1e and expressed as follows:

3 $$\theta \left(\alpha \right)=\arcsin \left(\frac{\lambda }{\Lambda }2\sin \left(\frac{\alpha }{2}\right)\right)$$

More detailed calculations are available in SI 1. In practice, we choose a wavelength at the threshold, *λ* = 1.01Λ, since a higher value would make order $\left(+1,-1\right)$ evanescent for large twist angles. Here, the maximum twist α is 60°, as seen in Fig. 1d; any higher α would see the order become evanescent. This is why Fig. 1e stops just before ${60}^{\circ }$.

The designs under study are divided into three distinct templates. The first template, illustrated in Fig. 1b treats the design as a stack of N mini-layers^22^. Each mini-layer’s dielectric profile is parameterized by three complex phasors (${z}_{1},{z}_{2},{z}_{3}$) defining the grating in Fourier space (see SI 1b for further details). The resulting signal is converted to a grating using thresholding with ${\varepsilon }_{{low}}=2$ and${\varepsilon }_{{high}}=4$. This representation leads to 6 free parameters plus an additional parameter for the mini-layer depth. A mini-layer parameterization thus resulting in 7 N parameters. The second and third templates consist of a set of N ellipses arranged in the ${xz}$ plane as illustrated in Fig. 1c. In the second template, each elliptical inclusion has a dielectric constant of $\varepsilon =4$, while in the third template, inclusions can take discrete ε values from 1 to 4. In both cases, the background permittivity is${\varepsilon }_{2}=2$. Each ellipse is characterized by its position, axes length and slant, resulting in 5N free parameters. Additionally, the layer thickness is a parameter, bringing the total to 5N + 1 parameters for the second template and 6N + 1 parameters for the third. Overlapping ellipses are combined using an OR operation.

The performance of each design ${\boldsymbol{D}}$ is evaluated based on the transmission value ${T}_{{ij}}$ of the targeted diffraction order (diffraction efficiency), averaged over twist angles from 0 to 60°, with *N* = 50 samples taken for the averaging. The figure of merit is thus a function of the design, diffraction order $(i,j)$, and twist angle

$$f\left({\boldsymbol{D}}\right)=\frac{1}{N}\mathop{\sum }\limits_{k=0}^{N-1}{T}_{{ij}}\left({\alpha }_{k};{\boldsymbol{D}}\right),{with}\,{\alpha }_{k}=k\frac{{60}^{\circ }}{N-1}$$

While we focus on the $(i,j)=(+1,-1)$ order, we will show that all conclusions can be adapted to the $(-1,+1)$ order through symmetry.

**Fig. 2:**
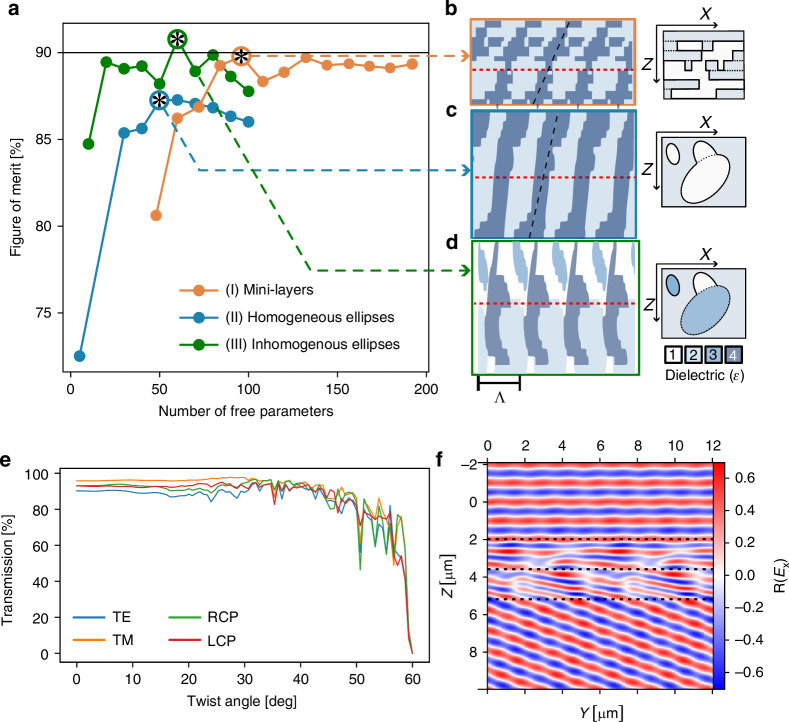
Numerical optimization results. **a** Performance of the different templates considered as a function of the number of free parameters. **b**–**d** Best devices obtained for the mini-layer, ellipses, and heterogenous ellipses templates respectively. The plane separating the two twisted photonic crystals is drawn in red. **e** The transmission in order $(+1,-1)$ for all twist angle values of the best design in the homogenous ellipse template (**c**). **f** The ${\mathfrak{R}}{{\rm{E}}}_{{\rm{y}}}$ field map for the previous design with Y polarization

Figure 2a shows the optimal figure of merit ${f}^{* }$ for each template relative to the number of free parameters, while Fig. 2b–d display the dielectric profiles for the best configurations of the three design templates. The optimal figure of merit ${f}^{* }$ is averaged across 4 polarizations (X, Y, RCP, and LCP). The mini-layers template reaches ${f}^{* }=90 \%$, slightly outperforming previous work (88%) that optimized the *X* and *Y* transmission^22^. This template produced devices very similar to those of previous work with half the number of mini layers. The ellipses design however provides simpler, more continuous structures yet still efficient, peaking just above 87%. This template was motivated by the tendency of the mini-layers template to produce filled structures roughly continuous along the *z*-axis, even across the twist plane. Compared to previous work, our ellipses design offers simplified fabrication and lower sensitivity to fabrication uncertainties, as will be discussed further down. When allowing the optimizer to choose between a discrete set of materials, the efficiency peaks at 91%, due to the greater freedom. With a fabrication procedure in mind, the parameterization can be constrained to match feasibility constraints, i.e., choosing from a pool of available materials for each component. This may be useful in two-photon polymerization (e.g., *Nanoscribe*^30^). While the latter offers lower refractive index contrast, it provides an attractive trade-off between structural complexity and process accessibility. Regardless of the template, the devices primarily consist of a roughly continuous slanted dielectric structure, tapering to thinner edges near the limits of the layers. When present, these thinner edges are improving the impedance matching with air, facilitating in- and outcoupling of light. While the optimal design for homogenous ellipses does not have this feature, it illustrates very well the slanted and continuous dielectric profile along z. As the number of free parameters increases, we initially observe an improvement of the performance. The performance however saturates and even decreases at some point for all templates due to two factors: the limited performance gain from a finer design and the intrinsic limitation of the optimizer to find the best solutions in an increasingly larger space. This limitation is present in all optimizers, known as the curse of dimensionality^31^.

Figure 2f shows how the best device in the ellipses template bends the incoming plane wave with a twist of 20°. More field maps for the complete range of the twist angle are available in S8.

As previously stated, Fig. 2 reveals a shared slant angle for template I and II, indicated by a dashed black line, confirmed by analysis of all optimized devices (see SI 2). This slant angle ranges from ${\gamma }_{{num}}=15$ to ${24}^{\circ }$. For the alternate diffraction order $(-1,+1)$, the optimal slant angle reverses, suggesting a fundamental relationship between slant angle and diffraction order. The following section justifies the existence of this slant angle using a structural blazing model.

**Fig. 3:**
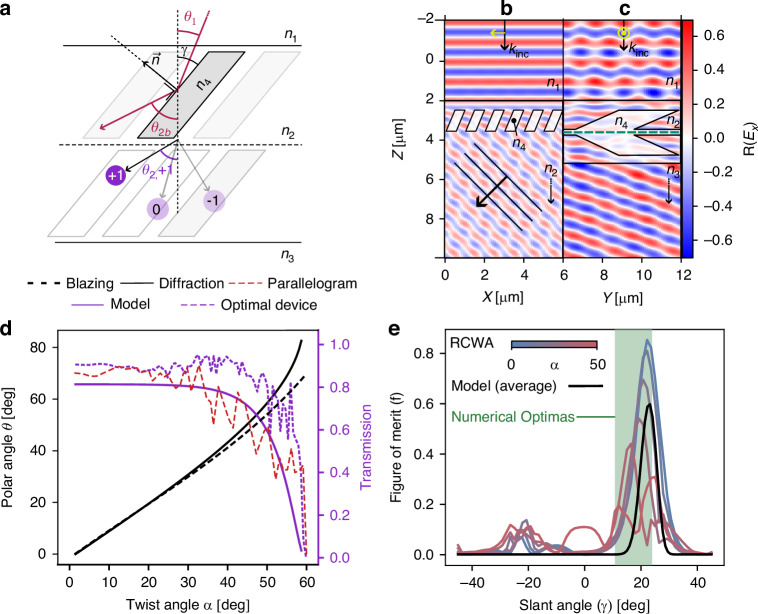
Analytical blazing model. **a** Scheme of the reduced structural model. **b** Field map for *Y* polarization with only one layer on a substrate of index ${n}_{2}$. **c** Field map for *Y* polarization with both layers twisted by an angle $\alpha =2{0}^{\circ }$ in air. **d** The diffraction angle from the grating Eq. (3) compared to the blazing angle formula (8). On a second axis, the diffraction efficiency is shown for our reduced model and RCWA simulations of optimized ellipses archetype and a parallelogram with the obtained optimal slant. **e** The figure of merit for different slant angles computed using our model compared to RCWA simulations for the different twist angles

The previous results highlight how various optimizations lead to shared features in the optimal designs. Specifically, devices exhibit a common slant angle that is dependent on the targeted diffraction order. From diffraction theory, the diffraction orders when going from a medium of index ${n}_{i}$ to ${n}_{i+1}$ are function of the periodicity of the grating, the wavelength, and the incidence angle (${\theta }_{i}$). This dependence is expressed by the gratings equation^32^

4 $${n}_{i+1}\sin \left({\theta }_{i+1,m}\right)={n}_{i}\sin \left({\theta }_{i}\right)-m\frac{\lambda }{\Lambda }$$

with $m\,{\mathbb{\in }}\,{\mathbb{Z}}$. While this equation predicts diffraction orders at discrete angles ${\theta }_{i+1,m}$, no clue is given of their intensity. Although specialized theories exist for the diffraction efficiency of common grating shapes^23,33^, solving Maxwell’s equations is required for the most general case. The structures studied in the present work fall under this general category, but the presence of a common slant angle makes them more closely resemble blazed gratings. Blazed gratings are gratings with a specific slanted profile that aims at concentrating the diffracted light in a specific diffraction order^24^. In this section, we prove that the slanted geometry is at the core of the device operation by applying blazing principles to a simplified structural model. The structural model simplifies the ${xz}$ profile of the unit cell used in each layer to isolate the blazing effect as illustrated in Fig. 3a. Each unit cell consists in a parallelogram of index ${n}_{4}=2$ on a background ${n}_{2}=\sqrt{2}$. The model discussed is valid for a wide range of parallelogram widths ($0.2\le w\le 0.8$) and heights (*h* > Λ). These dimensions have minimal impact on the subsequent analysis if the layers can be considered diffraction gratings. An illustration of their influence is available in SI. When comparing our model to RCWA in Fig. 3b–d, the height is fixed to 4.0 Λ and the width to 0.4 Λ. Again, without impacting the phenomenon discussed, we can consider the presence of a buffer interlayer of index ${n}_{2}=\sqrt{2}$ with a depth of 0.2 Λ between the two layers. This statement is supported by a numerical study in SI 9. The refractive indices in emergence and incidence media are kept the same (n_1_ = n*3* = 1) so that diffraction orders for the structural model can be analyzed by considering the same two successive diffraction events as for the optimized structures. The diffraction polar angles are still governed by Eq. 3, which is charted in Fig. 3d.

In the following development, we will treat the diffraction through each layer separately. The incoming beam is considered at normal incidence such that the incoming polar angle is ${\theta }_{1}={0}^{\circ }$, while ${\theta }_{2}$ is the orientation after diffraction through the first layer and finally ${\theta }_{3}$, the orientation in free space after the crystal which is given by Eq. 3. Starting with the upper grating, Eq. 3 indicates three propagative transmitted diffraction orders in the buffer layer ${n}_{2}=\sqrt{2}$, each with distinct emergence angles

5 $${\theta }_{2,m}=\arcsin \left(-m\frac{\lambda }{{n}_{2}\Lambda }\right)\approx -m\,{45}^{\circ }\,{with}\,{m}\in \left\{-1,0,1\right\}$$

We now analyze the power distribution among the available diffraction orders using a blazing heuristic. Blazing occurs in a specific order when the diffraction angle coincides with the specular reflection against the grating’s slanted surface. Similarly, blazing occurs with refraction through the same surface. From empirical analysis, blazing in the system occurs by reflection. This phenomenon is best illustrated by the field maps of Fig. 3b, where order *m* = +1 is seen blazing against grating fins oriented at γ = 22.5° for an outgoing wavevector at θ_2b_ = −45°. This field map is obtained by RCWA, deposing the upper, untwisted layer on a substrate of index n2 to isolate its diffraction properties. The observed outgoing polar angle closely aligns with the (+1) diffracted angle of Eq. 3, where ${\theta }_{2,+1}=-{45}^{\circ }$. Analytically, the blaze angle is derived from specular reflection against the surface slanted with an angle γ of the parallelogram illustrated in Fig. 3a

6 $${\theta }_{2,b}\left(\gamma \right)={\theta }_{1}-2\gamma$$

For blazing to occur, the blazing slant angle ${\gamma }_{b}$ is tuned to align the blazing angle (θ_2b_) with order +1

7 $${\theta }_{2b}\left({\gamma }_{b}\right)={\theta }_{2,+1}=-{45}^{\circ }$$

This relation is satisfied for a specific slant ${\gamma }_{b}={22.5}^{\circ }$. A value that aligns with the range of ${\gamma }_{{num}}=15-{24}^{\circ }$ from the previous numerical study.

The second grating, without being twisted (*α* = 0), has an identical blazing configuration to the first one. When describing the second layer, we consider that the first layer blazing is perfect: the output angle can be assimilated to the diffraction angle (θ_2_ = θ_2b_ = θ_2,_ _+ 1_). As the two layers share a common slant γ the blaze angle θ3b is expected at θ_2_ − 2γ = 0°. Consistently, the diffraction angle for this second diffraction event is obtained from Eq. 3 with an incidence at θ_2_ = 45° relative to the grating normal. At this angle, only order (−1) propagates, with θ_3,−1_ = 0°, equal to the blaze angle θ_3b_. The beam direction is therefore unchanged when traversing the untwisted bilayer through order $(+1,-1)$.

For a nonzero twist angle α, the model requires to consider the three dimensions of space as the orientation of the slanted surface of the grating depends on the twist angle α. This orientation is defined by the surface normal ${\boldsymbol{n}}\left(\alpha \right)$. The normalized outgoing wavevector ${{\boldsymbol{u}}}_{r}$ is obtained from the incident wavevector ${\boldsymbol{u}}$ on the second grating using a vectorial form of Eq. 6 commonly used in ray tracing^34^

$${{\boldsymbol{u}}}_{r}={\boldsymbol{u}}-2\left[{\boldsymbol{n}}\left(\alpha \right)\cdot {\boldsymbol{u}}\right]{\boldsymbol{n}}\left(\alpha \right)$$

The outgoing polar angle θ_3b_ is obtained by evaluating the angle between ${{\boldsymbol{u}}}_{r}$ and the layer normal ${{\boldsymbol{e}}}_{z}$ while accounting for refraction

8 $${\theta }_{3b}=\arcsin \left(\frac{{n}_{2}}{{n}_{3}}\sqrt{1-{\left({{\boldsymbol{u}}}_{r}\cdot {{\boldsymbol{e}}}_{z}\right)}^{2}}\right)$$

This blazing angle is compared to the diffraction angle in Fig. 3d for a slant angle *γ* = 22.5°. For small twist angles, the relation is mostly linear (below 30°), and the match between blaze and diffraction angles is excellent. For twist angles superior to 30°, the blaze and diffraction angles start to diverge. While this first formula indicates that the blazing will be less efficient for large twist angles, we do not yet have a clear picture on the impact of this divergence. The impact of misalignment on transmission values is modeled using a Gaussian distribution centered around the blaze angle:

9 $${t}_{i}\left(\theta \right)=A{e}^{-\frac{{\left(\theta -{\theta }_{{ib}}\right)}^{2}}{2{\sigma }^{2}}}$$

Here, the parameters A and σ are derived by fitting RCWA simulations of a single layer like the one on Fig. 3b. The standard deviation ($\theta =17^\circ$) quantifies the sensitivity of transmission to misalignment between blaze and diffraction angles. The amplitude A approximates the transmission coefficient for a slant angle in ideal alignment (∆θ = 0). This model assumes transmission approaches zero when the blaze and diffraction angles are significantly misaligned. The resulting transmission is given by

10 $$t={t}_{1}\left({\theta }_{2b}-{\theta }_{2}\right){t}_{2}\left({\theta }_{3b}-{\theta }_{3}\right)$$

which accounts for transmission losses due to misalignment in both layers. This approximation is compared in Fig. 3e to RCWA simulation for the optimal device in the ellipses template across varying twist angles. Our model respects the profiles observed during optimization and successfully predicts the steep downward trend between 45 and 60°. The optimized device used for comparison improves upon the reduced model over the whole control range. The performance of a device consisting in a single parallelogram is finally charted in dotted red line, more closely matching the analytical model. Compared to our reduced model, optimized designs tend to use curved surfaces along z, unlike our parallelograms, offering a distribution of slant angles γ(z). Although it is not the case for the best homogenous ellipses design, designs exhibit thinner higher dielectric regions as we get close to the outer surface of the layers. This phenomenon is due to impendence matching, much like in light extraction optimizations in light-emitting diodes inspired by fireflies^35,36^. It is noteworthy that the blazing behavior alone in this simple structure yields a figure of merit around 70%. The field map in Fig. 3c shows the reduced device in operation at $\alpha ={20}^{\circ }$, with, indeed, a convincing plane wave going out of the device. The optimizer then further enhances this blazing effect, even for a single homogenous ellipse, leading to 73% efficiency. This result is coherent as the improvement, for a relatively similar device complexity, comes mainly from the fine-tuning of the orientation and size of the dielectric inclusion. The model’s figure of merit—defined in Eq. 2 as the average transmission over the full range of twist angles—is plotted for various slant angles in Fig. 3e. The results show a clear optimum at $\gamma \approx {23}^{\circ }$, consistent with previous numerical predictions. As a benchmark, RCWA-computed figure of merit for this reduced model is also shown for a set of twist angles. Interestingly, it confirms that this blazing behavior applies uniformly across twist angles ranging from 0 to 30°. The optimal slant γ seems to decrease with increasing twist α, supporting the hypothesis that a distribution γ(z) is indeed beneficial to optimized designs. This result also suggests that specific devices could be optimized, targeting large twist (and hence polar) angles specifically. The stability of the blazing behavior is key to the device operation as we already get a figure of merit around 65% for this reduced model. The main loss of efficiency is for large twist angles, the reduced model offers a great avenue for fabrication. This simple parallelogram shape can provide efficient beam steering in the 0–30° twist range as shown in Fig. 3d. Manufacturing could be more easily applied using electron beam lithography and tilted etching^37^.

The model does not capture the smaller RCWA peaks in Fig. 3e, around 0° and $\gamma \approx {20}^{\circ }$ mainly due to the assumptions in Eq. 9. Indeed, we neglect blazing through and against the other surfaces of the parallelogram as well as blazing that also happens on the other (incidence) side of the crystal. Guide mode resonances can also make this efficiency noisier by introducing sharp variations in the transmissions (more details in S6). However, these phenomena are of lesser magnitude, allowing our model to successfully explain the behavior of the crystal. We conclude that this structural model of the identified blazing behavior underscores the importance of the blazed configuration.

**Fig. 4:**
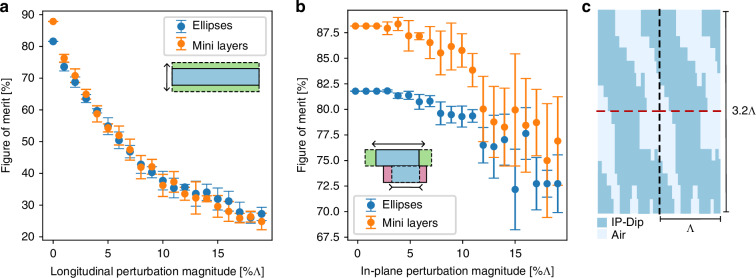
Impact of Parameter Uncertainty and Fabrication Tolerances. **a** Sensitivity of the figure of merit for different perturbation magnitudes of the layer depths. Perturbations are considered as a percentage of the pitch $\Lambda$ **b** Sensitivity of the in-plane features to perturbations. **c** Design proposition specific to two-photon polymerizations using the ellipses archetype ($N=12$) and IP-DIP material, a high-refractive-index, negative-tone photoresist tailored for two-photon polymerization

Before concluding this article, we consider the experimental feasibility of the proposed devices. First, sensitivity of the optimal design to deformations of the dielectric distribution is evaluated in Fig. 4. Figure 4a evaluates the sensitivity of an intricate mini-layers design with $N=22$ layers and an ellipses design with $N=12$ to longitudinal (depth-wise) perturbations. The perturbations are variations of the layer’s depth with amplitude following Gaussian noise. Both mini layers and ellipses react strongly to these kinds of perturbations, losing 30% efficiency for 5% variations amplitude. We observed that this behavior is mainly due not to the loss of blazing but rather on the diminishing transmission of the effective-index bilayer. Indeed, higher perturbations have a chance of significantly thinning or thickening the bilayer, resulting in more reflected power. As a countermeasure, we propose to deposit a homogeneous anti-reflective coating of tailored depth after fabrication^38^.

Figure 4b shows how the mini layer and ellipses devices react under in-plane erosion or dilation of high dielectric constant regions. This dilation or erosion represents defects that can occur during etching or 3D printing. From this figure, we conclude that the intricate mini layers are more sensitive to in-plane perturbations. Overall, these perturbations are less impactful than the layer depth. For C-band optical fiber optics (1550 nm) 5% error signifies uncertainty of 77 nanometers on the thickness of the layers. Even if the ellipses archetype is shown more robust, with less variations of the performance under imprecision, we still would no dismiss intricate mini-layer designs. Even with great errors, particularly in-plane ones, mini layers are still better performing. In summary, depending on the capability of the fabrication apparatus, ellipses designs are more reliable, and mini layers have a higher performance ceiling but are pickier on design parameters. *Nevertheless*, Fig. 4b*shows that both devices are stable with up to 5% planar perturbations while depth-wise perturbations should never reach 5% but more likely cater to less than 2% (31* *nm)* in the optical range.

In the gigahertz range, our structures can be manufactured using ceramic resins for stereolithographic (SLA) printers^39^. These resins can be processed accurately from 25 to 100 microns depth-wise and 300 microns in-plane. Here, even the most complex structures can be manufactured reliably up to the K band (18–27 GHz) with corresponding pitch $\Lambda =1.67-1.11{\rm{cm}}$. In the optical range, electron beam lithography with tilted etching^37^ is adapted to our simplified devices of the ellipses template and the parallelogram reduced model. Given the slanted profiles we identified, tilted etching could reduce the need for membrane transfer. Ideally, a single mask could be used to produce a parallelogram device. We finally propose an alternative design path in the optical range using two-photon polymerizations with an efficiency of 79% in Fig. 4c. In this design, the structure is porous, and the highest dielectric constant is 2.62 (resin IP-n162). This approach has the advantage of a great freedom regarding the pattern to print as well as the possibility of a porous device, enabling to still have enough contrast for the device operation.

Scanning LIDAR technologies would greatly benefit from all-dielectric tunable beam steering. The LIDAR beam can move around the area to scan, increasing spatial resolution and diminishing the power required. The beam orientation could be controlled by rotating the two crystal slabs using MEMS[*]. In telecommunication, beam steering could also improve energy efficiency, especially for satellite communication, where tracking moving receivers is necessary. This is currently done using moving antennas. Here we offer a solution when the motion lies in a planar device. For telecoms, it could take a compact form: two resin/ceramic disks of a few centimeters rotating azimuthally. The device has no limit on the in-plane dimensions, one can benefit from the high detection area. Other applications include laser processing/surgery and augmented/virtual reality. We think the proposal of a concrete implementation will be possible in future work and is already viable in the gigahertz range.

## Discussion

Firstly, our study demonstrates that heuristic optimization enables the rapid design of high-efficiency beam-steering devices based on twisted bilayers photonic crystals. Mini-layered structures achieved up to 90% beam steering efficiency with twice as few layers as previous work^22^. Another, more organic template achieves a slightly lower efficiency (87%) but offers a simpler design. Similarly, solutions based on discrete material choices offer enhanced (91%) performance. Though slightly less manufacturable than the ellipses design, they still present promising prospects for practical fabrication. Secondly, we found that the optimal devices rely on a bilayer blazing effect, where a specific slant angle allows sequential blazing into the (+1) and then (−1) orders of the first and second layers. The change in twist angle alters the reciprocal space geometry, adjusting beam orientation while preserving the blazing condition for twists up to 45°. We proved using a reduced model that this preservation of the blazing condition is twisted photonic crystals is excellent. For small twist angles, the diffraction and blazing angles are predominantly linear and align perfectly. The model also predicts the steep decrease in diffraction efficiency for large twist angles and identifies an optimal slant angle of approximately ${23}^{\circ }$. Optimized devices produce diffraction efficiencies consistent with this model but achieve higher overall performance. These devices consistently feature slant angles close to the model’s predicted value. The performance enhancement appears to result from two key mechanisms: impedance matching through engineering of the refractive index distribution and variation of the structure’s slant angle using curved geometries along the crystal’s normal direction. From our simplified model, we propose a last, very simple design that sees the cross-section of the device the shape of a parallelogram. This design proves particularly efficient in the small twist angle range from 0 to 30°, as shown in Fig. 3d.

## Materials and methods

The numerical setup that produces optimal designs is composed of two parts. First, extended rigorous coupled wave analysis (RCWA) is used to solve Maxwell’s equations for the designs^40^. Unlike classic RCWA, which requires twist angles that form a commensurate superlattice, this extended RCWA allows for the numerical characterization of twisted bilayers at any twist angle. By avoiding the supercell approach, this extension enables efficient (fast) numerical experiments by selectively computing a subset of scattering matrix coefficients. Second, an optimizer explores candidate designs in the parameter space. In particular, surrogate-assisted particle swarm optimization (PSO) is employed^28,29^. The surrogate model significantly reduces the number of RCWA simulations needed by leveraging faster predictions from a neural network model, as in refs. ^41,42^. Particle swarm optimization is particularly suited to continuous geometric parameters, but this implementation also handles categorical parameters. For example, it allows for material parameterization in the third template. Particle swarm optimization requires the definition of a figure of merit for the diffraction efficiency to sort the designs from worst to best. The combination of extended RCWA with PSO facilitates rapid computation (2 s for $$N=60)$$ of this figure of merit in embarrassingly parallel workloads (16 threads of a Rome 2.9 Hz CPU). Therefore, 1000 devices were designed for each of the three different parameterization templates. In total, 4.5 million devices were evaluated, among which 450 thousand were simulated.

## Supplementary information

Supplementary material for Twist-Induced Beam Steering and blazing effects in photonic crystal devices

## Data Availability

The RCWA package used in this work is available on the Khepri GitHub page https://github.com/Kaeryv/Khepri. The Keever framework and Hybris PSO optimizer are available here https://github.com/Kaeryv/Keever and here https://github.com/Kaeryv/Hybris. Specific codes used for launching simulations and generating figures of this article are available in a separate repository. The data that support the findings of this study are available from the corresponding author upon reasonable request. Supplementary information accompanies the manuscript on the Light: Science and Applications website (http://www.nature.com/lsa).
