# Supplementary material for: Twist-Induced Beam Steering and Blazing Effects in Photonic Crystal Devices
Source: Light Sci Appl. 2025 Aug 7;14:263. doi: 10.1038/s41377-025-01942-7 (PMC12331998; doi:10.1038/s41377-025-01942-7)
Supplement: Supplementary file 1 — Supplementary material for Twist-Induced Beam Steering and blazing effects in photonic crystal devices [file 41377_2025_1942_MOESM1_ESM.pdf]

Supplementary information for Twist-Induced Beam Steering and Blazing Effects in Photonic Crystal Devices

S1: Obtaining the 2D grating equation

The g-vectors for twisted layers are given by rotating the basic lattice on which both crystals are built:

$$\{g_1\} = \left\{R(\alpha_1)i\frac{2\pi}{\Lambda}\vec{u}_x \ \forall i \in \mathbb{Z}\right\} \text{ and } \{g_2\} = \left\{R(\alpha_2)j\frac{2\pi}{\Lambda}\vec{u}_x \forall j \in \mathbb{Z}\right\}$$

with

$$R(\alpha) = \begin{bmatrix} \cos(\alpha) & -\sin(\alpha) \\ \sin(\alpha) & \cos(\alpha) \end{bmatrix}.$$

The resulting reciprocal space for the twisted bilayer is obtained using element-to-element sum or Minkowski sum

$$\{g_1 + g_2\} = \left\{(i+j)\cos\frac{\alpha}{2}\frac{2\pi}{\Lambda}\vec{u}_x + (j-i)\sin\frac{\alpha}{2}\frac{2\pi}{\Lambda}\vec{u}_y \ \forall i,j \in \mathbb{Z}^2\right\}.$$

For specific order $(i,j) = (+1,-1)$, we obtain this for instance.

$$g_y = -2\frac{2\pi}{\Lambda}sin(\frac{\alpha}{2}).$$

This particular order has a twist-dependent value.
The outgoing polar angle $\theta_{3,+1,-1}$ is obtained using the projection g_y of the outgoing wavevector on the the crystal surface

$$g_y = k_y = k_0\sin(\theta) = -2\frac{2\pi}{\Lambda}sin\left(\frac{\alpha}{2}\right)$$

$$|\theta| = \arcsin\frac{\lambda}{\Lambda}2\sin\frac{\alpha}{2}$$

S1 b: Gratings defined in Fourier space

In the mini-layers template, each mini-layer is a grating with a dielectric constant depending on one dimension x. There are several ways to define this function. We can for instance define the grating $\varepsilon(x)$ as a union of rectangular pulses $r(x,\bar{x},dx)$ of width dx and center $\bar{x}$

$$\varepsilon(x) = \max\left[r(x;\bar{x}_1,\Delta x_1), r(x;\bar{x}_2,\Delta x_2), r(x;\bar{x}_3,\Delta x_3)\right],$$

with

$$r(x, \bar{x}, dx) = \begin{cases} \varepsilon_{\text{high}}, & \text{if } \bar{x} - \frac{\Delta x}{2} \leq x \leq \bar{x} + \frac{\Delta x}{2}, \\ \varepsilon_{\text{low}}, & \text{otherwise.} \end{cases}$$

While this first approach is enough for defining gratings, it does not naturally account for the periodicity of the unit cell: it is not guaranteed that $r(x = \Lambda) = r(x = 0)$. The variety of patterns is also quite low for three rectangular pulses and $N = 6$ free parameters.
A parameterization that lead to better results uses Fourier space to define the grating. A continuous function $f(x)$ is decomposed in a truncated Fourier series

$$f(x) = \sum_{i=1}^{3} \Re z_i e^{i2\pi w_i x^*} = \sum_{i=1}^{3} A\Re e^{i2\pi w_i x^* + \phi_i}$$

with $x^* = \frac{x}{\Lambda}$, the reduced x coordinate and w_i half-integer frequencies. The three complex parameters z_i lead to 6 parameters due to the real and imaginary parts. By property of complex numbers, these can be seen as the amplitude and phase of harmonics. The final expression for ε is obtained by thresholding.

$$\varepsilon(x) = \begin{cases} \varepsilon_{\text{high}}, & \text{if } f(x) \geq 0, \\ \varepsilon_{\text{low}}, & \text{otherwise.} \end{cases}$$

By considering the three first harmonics, a wide range of gratings can be generated. It is possible to use more harmonics but these tend to become less impactful as associated new features become smaller and smaller.

S2: Global analysis of optimized devices

Fig. S2 represents all the designs we obtained during optimization using U-Map `mcinnes2020umap` projection overlayed with agglomerate clustering. S2 also presents the average design is each cluster.
This discriminative analysis does indicate a slight segregation between the designs. However, the large majority of designs share a similar profile with a slant angle that is illustrated in the rightmost averaged design. The slant angle ranges from $\gamma_{\text{num}} = 15^\circ$ to 17°. When optimizing for the other available diffraction order $(-1, +1)$, the optimal slant angle is the exact opposite. This suggests a fundamental reason behind the slant that is linked to the diffraction order.
In the following section, the existence of the slant angle is justified by a structural blazing

model of the structure.

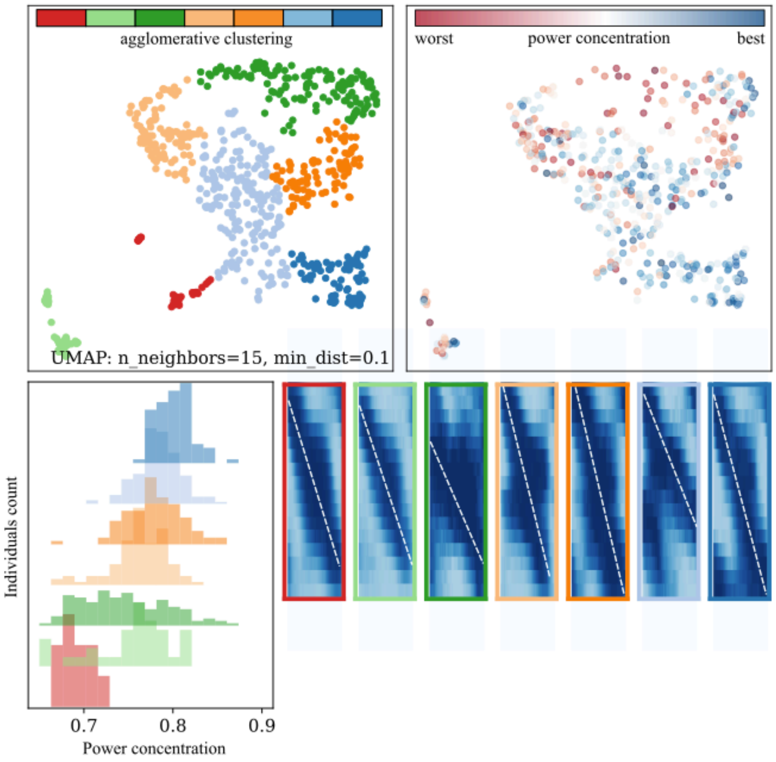

> **Figure S2** (upper left) U-Map projection overlayed with agglomerative clustering of the dielectric profiles of the optimized devices. (upper right) The same U-Map projection with the fitness (diffraction efficiency) overlayed. (below left) Average dielectric profile of each cluster sorted by performance, from left (worst) to right (best). In the bottom right figure, the identified slant is highlighted in white for each mean dielectric distribution of each cluster.

S3: Influence of height and width

Figure S3(B) shows the dependency of the blazing phenomenon on the height and slant angle. For very tall parallelograms, the blazing is sharper while for very small ones, blazing disappears. It is logical as heights near the wavelength $\lambda \approx \Lambda$ make the scatterer geometry less impactful in scattering.
Figure S3(C) also shows how this dependency is preserved for various twist angles by illustrating the transmission contrast

$$C = \frac{T_{+1,-1} - T_{-1,+1}}{T_{+1,-1} + T_{-1,+1}},$$

between orders (+1,-1) and (-1,+1). When $C = 1$ (purple), all transmission occurs in (+1,-1) order while C=-1 indicate transmission in the (-1,+1) order. A breakdown of the contrast in

transmission between orders (+1,-1) and (-1, +1) occurs for twists above 30 degrees.

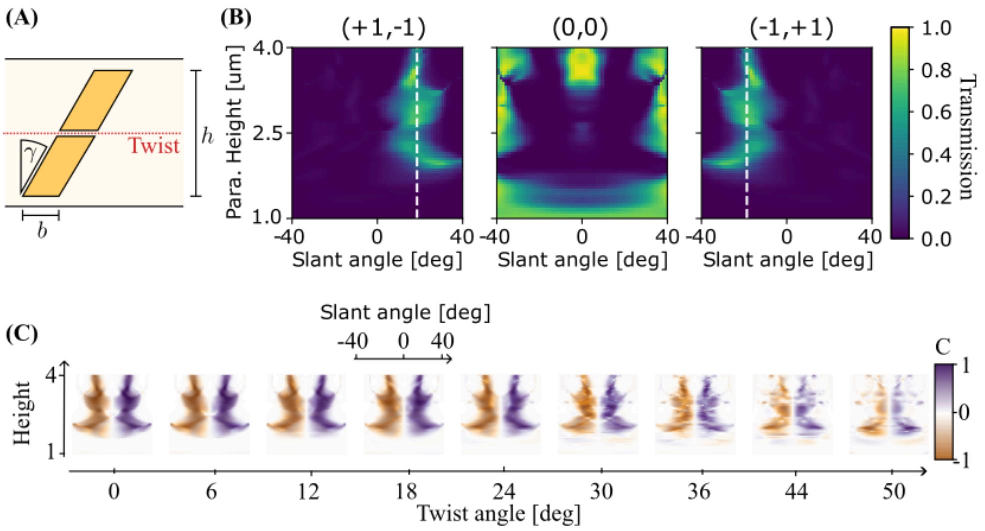

Figure S3 (A) Schematic of the reduced model. (B) Dependence of the transmission of the height and slant of the parallelogram for the three propagative diffraction orders. (C) Evolution of the contrast C between orders (+1,-1) and (-1,+1) for a range of twist angles.

S4: Other diffraction orders

Figure S4 is similar to S3(B) but presents the dependency on the width rather than the height of the parallelogram. Here, it appears than the width shall be not too wide or too thin (relative to Λ).

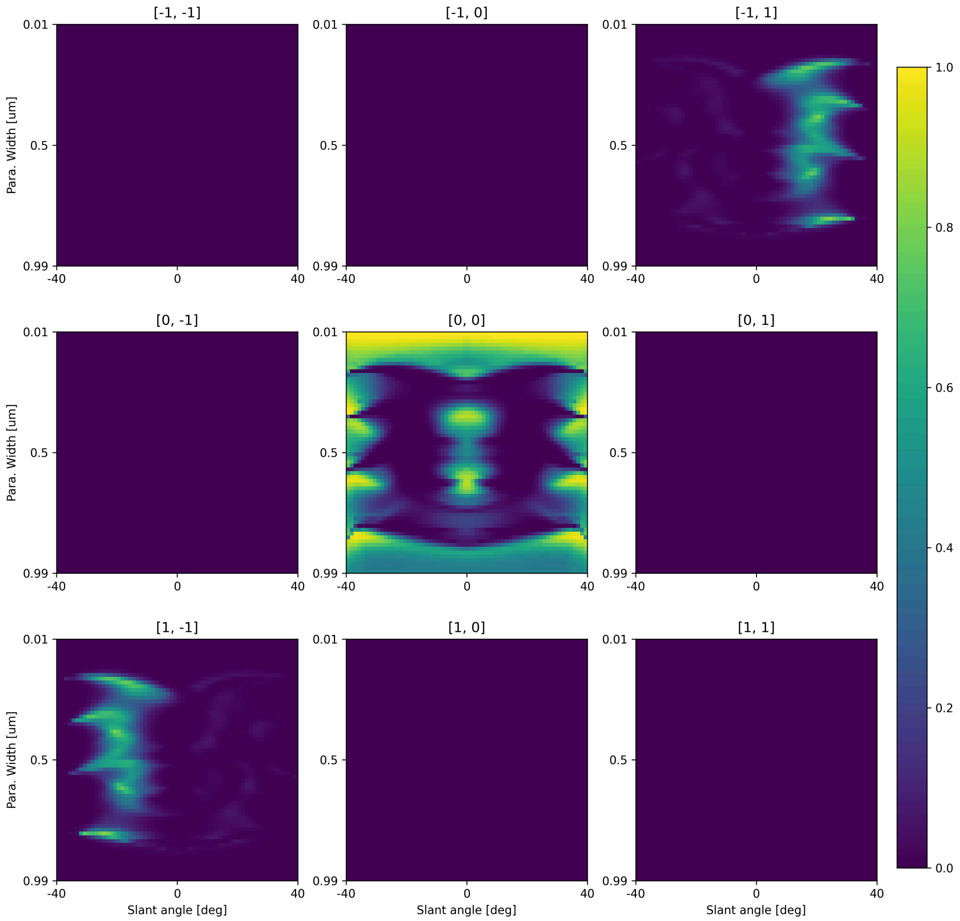

> **Figure S4** Dependency of the transmission on the parallelogram width and slant angle for 9 diffraction orders computed using RCWA. The slant angle on the abscissa goes from -40 to 40 degrees and the parallelogram with goes from 0.01 to 1 um.

S5: Single-layer operation of the optimized device

We illustrate in **S5** how only the first PhC slab (first half of a device) operates. This case uses an optimized devices. The slab is present between the two red lines.

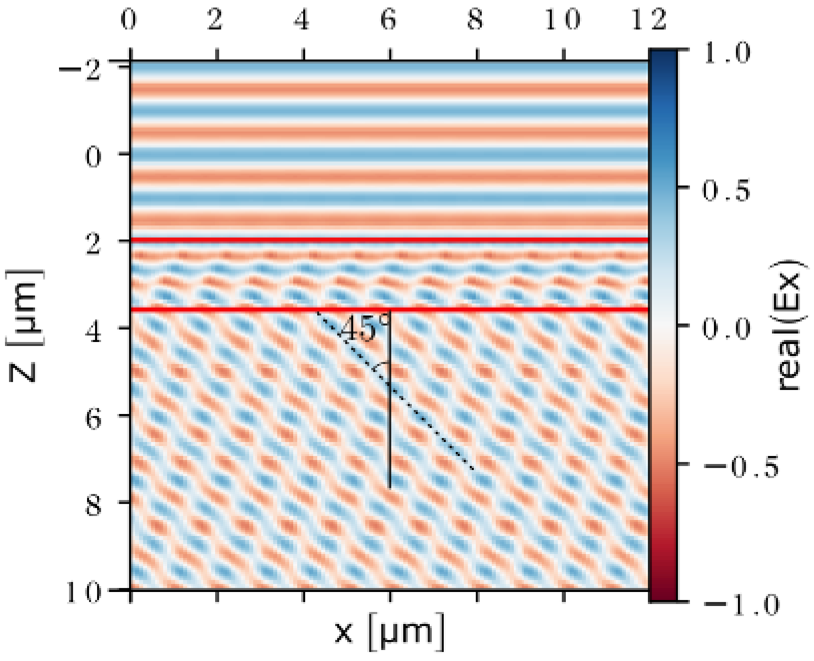

> **Figure S5** Electric field map of the X-polarized plane wave incident upon only the first half of the optimized ellipses template $N = 12$.

S6: Frequency sensitivity

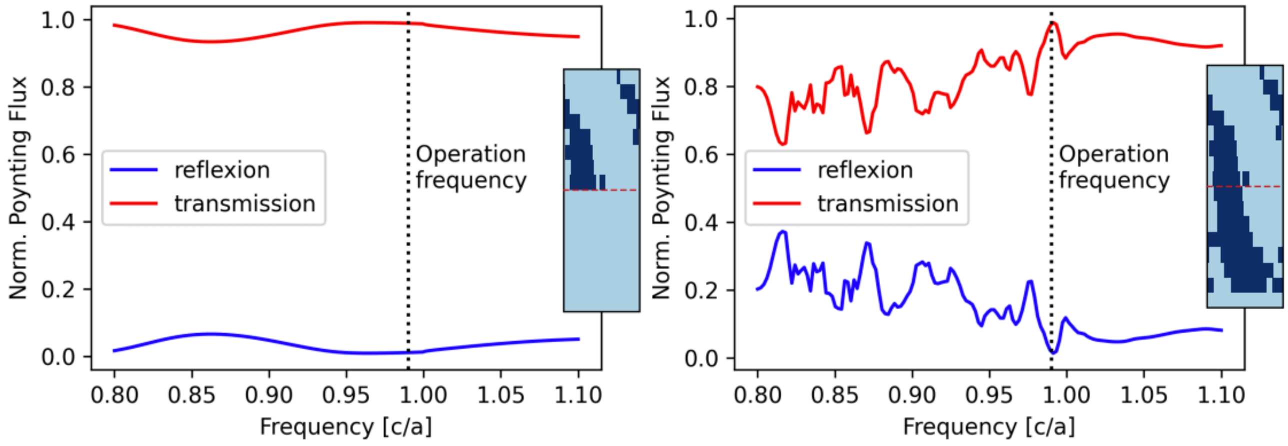

> **Figure S6** Spectra showing resonant frequencies of (A) a single layer (B) both layers. We observe that a single layer does not host GMR in the frequency range of interest.

The operation frequency is determined by our *evanescence condition* $\lambda = 1.01\Lambda$ which in turn causes the absence of guided mode resonances in a single layer around operation

frequency at normal incidence. The GMRs may however still happen in the bilayer, as the second layer sees a wave incoming at an angle of 45 degrees. While we did not setup priors in our optimization to counter these GMRs, the optimizer will try to exploit them to maximize transmission. This very fact is visualized in a transmission spectrum now shown in supplementary S6, reported here in Figure 4. This Figure shows how the twisted bilayer transmission pattern is complexified. While it is difficult to identify specific guided modes, we see that the optimizer manages to exploit a peak in transmission at the design wavelength. The behavior of a single layer can also be seen in the field maps of S5.

S7: Numerical analysis of the parallelogram under different twists

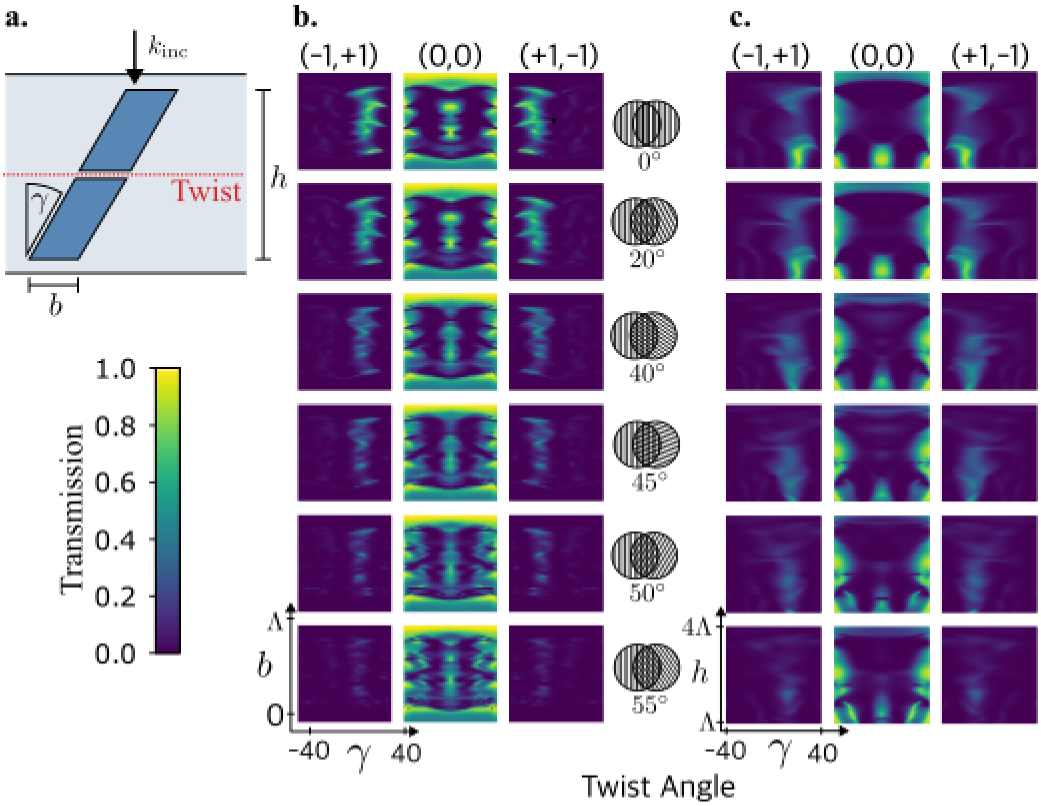

> **Figure S7** Extensive analysis of the transmission inside the other diffraction orders. We observe the dependency of T on parallelogram width w, basis b and the twist angle α.

S8: Behaviour of the fields at any twist angle

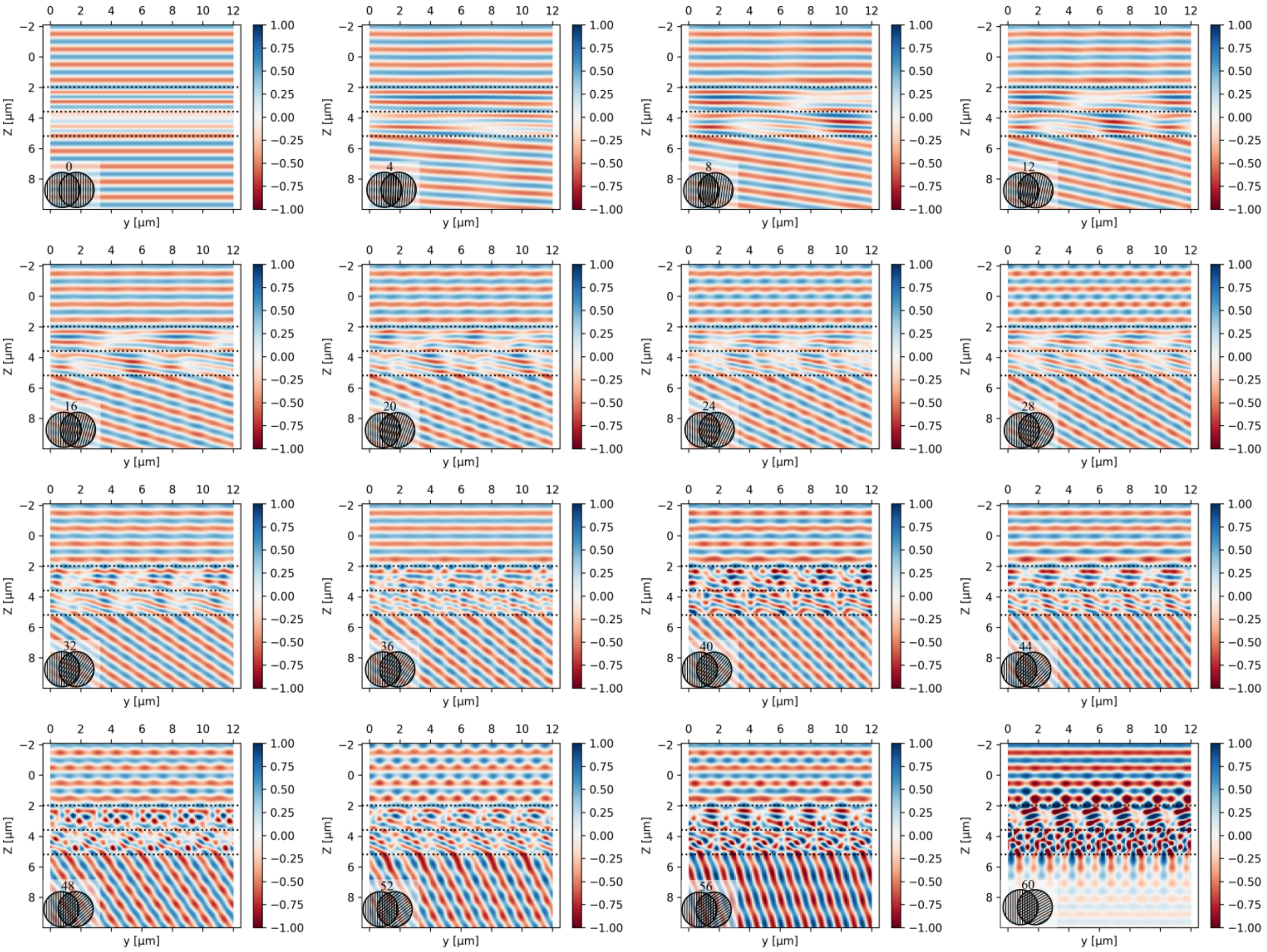

Figure S8 Field maps E_x for different twist angles α. The structure used is the optimal ellipses template ($N = 12$). We note in particular the evanescent fields in emergence medium for $\alpha = 60$.

S9: Effect of an low-index interlayer

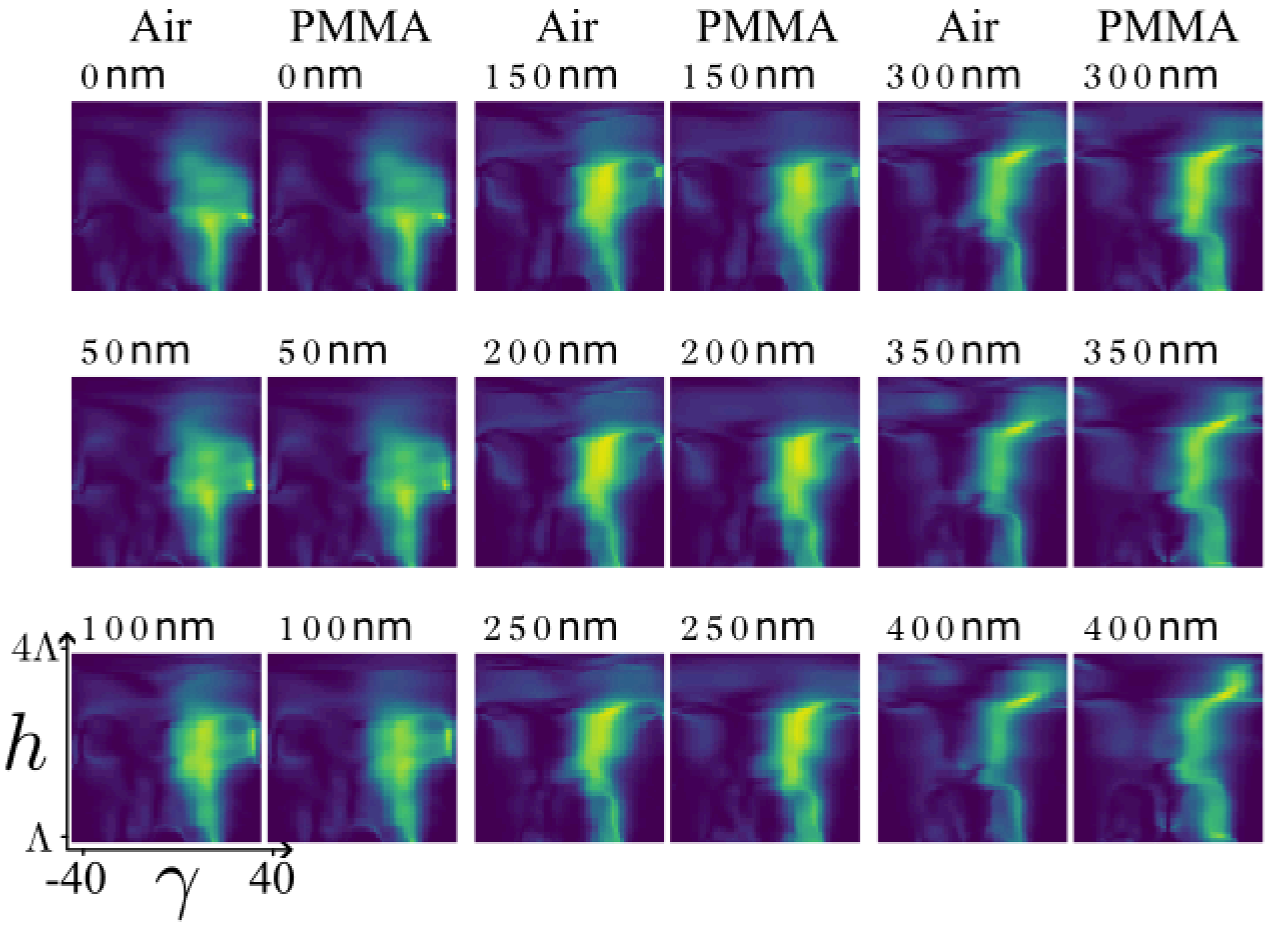

Figure S9 Effect of the addition of a PMMA / Air interlayer of growing depth.

We provide in S9 an illustration of interlayer effects on the parallelogram model. We see how the dynamic, while slightly affected by the addition of the lower index layer is not in any way destroyed or inoperative. This is expected as an intermediate uniform medium should not intervene in the geometry of diffraction. Only a slight influence on efficiency is produced from the moment there is an additional interface.

Systematic in-plane errors impact on figure of merit

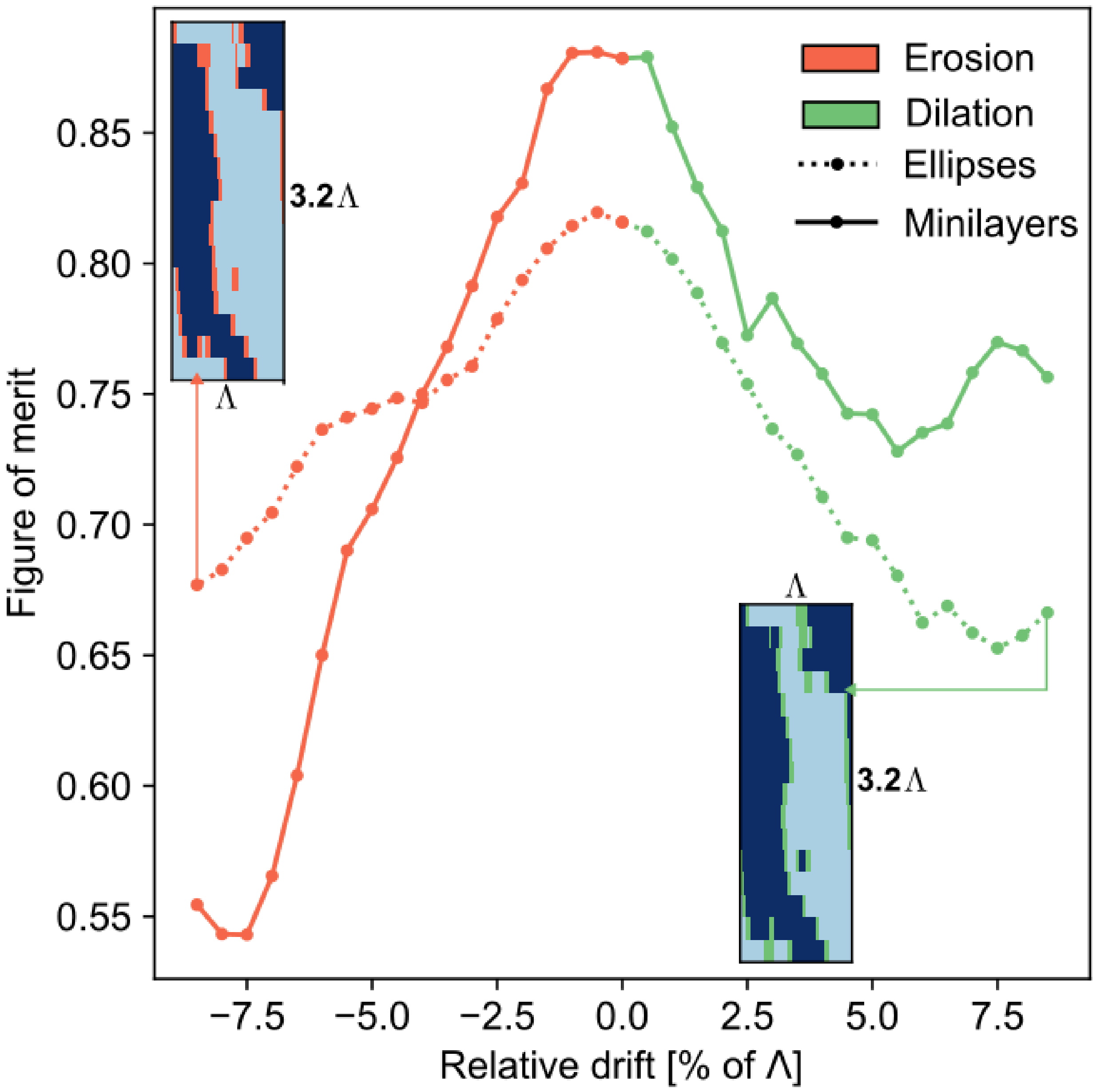

Figure S10 Sensitivity of the figure of merit regarding a systematic erosion or dilation of all layers. The dependency is shown for a mini layer and an ellipses archetype.

Figure S10 shows that a systematic error is extremely detrimental to the device performance. The ellipses template is more robust but still sensitive. Hopefully, this kind of error can be compensated by careful calibration (e.g. every etched pattern is too large).
